# Modelling extensions for multi-location studies in environmental epidemiology

**DOI:** 10.1177/09622802241313284

**Published:** 2025-02-05

**Authors:** Pierre Masselot, Antonio Gasparrini

**Affiliations:** 1 Environment & Health Modelling (EHM) Lab, Department of Public Health, Environments & Society, 4906 London School of Hygiene & Tropical Medicine, UK

**Keywords:** Environmental epidemiology, meta-regression, spatial interpolation, time series

## Abstract

Multi-location studies are increasingly used in environmental epidemiology. Their application is supported by designs and statistical techniques developed in the last decades, which however have known limitations. In this contribution, we propose an improved modelling framework that addresses these issues. Specifically, this flexible framework allows the direct modelling of demographic differences across locations, defining geographical variations linked to multiple vulnerability factors, capturing spatial heterogeneity and predicting risks to new locations, and improving the assessment of uncertainty. We illustrate these new developments in an analysis of temperature-mortality associations in Italian cities, providing fully reproducible R code and data.

## 1 Introduction

Multi-location studies have become the standard in environmental epidemiology due to the need to investigate health risks across regions and populations. Pooling several locations together increases the power of environmental epidemiology studies, allowing for the accurate estimation of the impacts on health from various exposures.^1–7^ This is helped by the increasing availability of global^8,9^ and high-resolution^10,11^ datasets for many environmental stressors.

The standard analytical approach for multi-location studies is the two-stage framework,^12,13^ which consists of the following steps: (i) location-specific associations between an environmental exposure and a health outcome are independently estimated, and (ii) these associations are pooled in a second-stage meta-analysis. This approach, developed in the last decades, is still an active research topic resulting in flexible two-stage designs.^14,15^ The two-stage analytical framework, in these settings, presents computational convenience compared to alternative single-stage multilevel models such as Bayesian disease mapping or Poisson log-linear models.^16–19^ In a longitudinal context typical of environmental epidemiology, the first stage allows a fine characterisation of complex exposure-lag-response relationships through modern techniques, while controlling for trends and time-varying confounders specific to each location.^20^ The second stage then allows improving first-stage estimates of association both at the pooled and location-specific levels, as well as allowing the assessment of heterogeneity and its drivers. Modelling this complexity through single-stage methods would necessitate an intractable number of parameters to estimate, and single-stage studies in some applications necessitate simplifications of the models, with potential biases in the estimates.

Despite its inherent advantages, the current two-stage analytical framework still presents limitations that hamper the generalisation and accuracy of its results. First, in multi-country studies, the use of administrative data with inconsistent stratification prevents sub-group analyses from assessing differential risks within a population, for example by age. This ultimately hampers the comparability of burden estimates between countries, or more generally between populations with very different structures. This creates confusion as to whether differential impacts are due to local vulnerabilities or to varying age structures across populations. Second, differences in risks across locations can be due to complex patterns involving several drivers of vulnerability, usually highly correlated with each other. Unfortunately, the computational complexity and limited sample size of meta-analytical models limit the integration of these drivers in the second stage to only a handful of location-specific characteristics, posing important limits to the amount of heterogeneity that such models are able to capture.^21^ Finally, the computation of the uncertainty in the impact measures at various geographical levels is made difficult by the correlation of location-specific risks estimates induced by the second-stage meta-analytical procedure. It is important to account for the uncertainty at the various stages of the analysis to avoid an underestimation of the uncertainty, especially in the analyses involving a high number of small location-specific datasets and low heterogeneity.

This contribution describes specific modelling extensions of the two-stage framework for the analysis of environment-related health risks, specifically addressing the limitations highlighted above. This extended framework allows for estimating health burdens in populations with little or no health data available,^22,23^ risk disaggregation for coarsely and inconsistently grouped populations, in particular by age groups,^22^ or spatial downscaling of the health risk.^24^ We additionally expand this risk prediction framework to the important step of health impact assessment, notably through age-standardisation of the burden estimates and accurate uncertainty assessment. The framework is structured in eight separate sections introducing analytical developments at various stages. Each section represents a package that can be considered or discarded depending on the objective of the analysis using this framework. It is illustrated through a case-study application using a real multi-location database of Italian cities. The example application is fully reproducible using code and data freely available in an online repository.

## 2 Data

The case study demonstrates an application of the framework in an analysis of temperature-mortality associations in 87 Italian cities within the period 2011 to 2021. The list of cities represents a sub-sample of a large urban database defined by Eurostat.^25^ To illustrate one of the extensions, specifically related to the prediction of the risk to new locations, we hold out the data for 27 (30%) of the cities, considering them as *unobserved* and assuming that no mortality data is available to directly estimate temperature risks. The list of cities and specific details are available in supplementary materials.

Each city includes series of daily mortality counts and mean temperature. Mortality series are extracted from a dataset made publicly available by the Italian National Institute of Statistics (ISTAT) that includes all deaths from 7904 municipalities across Italy.^26^ We construct city-specific series by aggregating deaths for any cause from municipalities within the selected cities for five age groups (0–44, 45–64, 65–74, 75–84 and 85 and older). Temperature series are extracted from the fifth generation of European Reanalysis (ERA5)-Land dataset, which provides daily mean temperature at 2 m on a global grid of approximately 9 km.^8^ Series are constructed by averaging pixel centroids contained within city boundaries provided by Eurostat.

We also defined 21 city-specific meta-variables representing socio-economic, land-use, environmental and climatic factors, extracted from the Eurostat urban and regional databases as well as from various reanalysis and remote sensing products. These 21 meta-variables are used to characterise differences in local vulnerability. We extracted death rates, population size and life expectancy for the five age groups in each city from Eurostat. These statistics are used to compute baseline mortality series and impact measures. Finally, we also extracted point locations for each city from OpenStreetMap data. The full list and definitions are provided in supplementary materials and provided in previous work.^22^

## 3 Methodological framework and results

**Figure 1.**
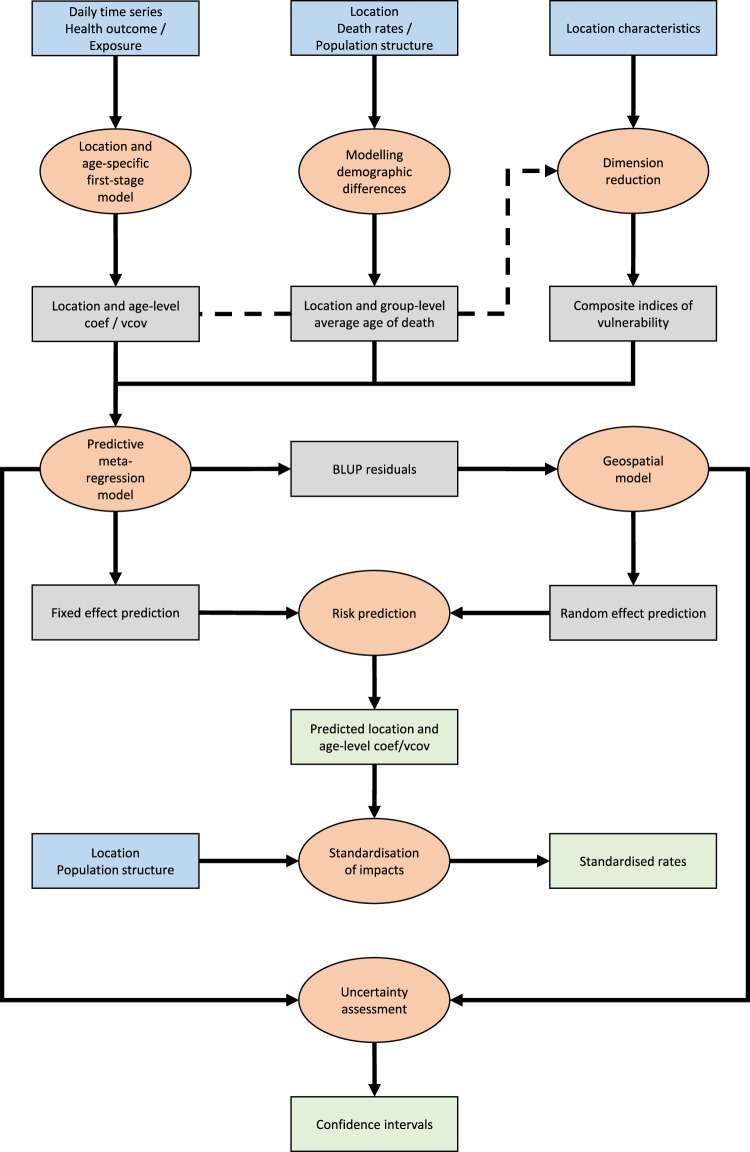
Flowchart describing the eight steps of the predictive framework. Orange ellipses indicate the eight steps, while rectangles indicate data, with blue being input data, grey being intermediate data and green being results. For color figure, see online version.

In this section, we present eight main steps of the extended methodological framework, each as a self-contained package with its own section. Relations between packages and the workflow of the analysis are shown in Figure 1. Within each section, we introduce the methodological details, the specific choices made for the temperature-related mortality application and the results of this application. The structure is replicated in the R scripts reproducing the analysis.

### 3.1 Location- and age-specific first-stage model

#### 3.1.1 Motivation and description

The first step of the methodological framework is to estimate location- and age-specific first-stage associations between the exposure and health outcome of interest where available. Generally, the objective of the first-stage model is to obtain an estimation of a vector of parameters $\theta_{ia}$ that defines the association for age group *a* within location *i*, along with its variance–covariance matrix $S_{ia}$ . Note that $\theta_{ia}$ can be further disaggregated by sex or any other individual characteristic, although we focus on age groups in the following. The first-stage model can generally be defined as:

(1) $$g(Ey_{iat})=\alpha_{ia}+f(x_{it},l;\theta_{ia})+\overset{J}{\underset{j=1}{\sum }}\;s_{j}(t;\varphi_{iaj})+\overset{Q}{\underset{q=1}{\sum }}\;h_{q}(z_{iaqt};\gamma_{iaq})$$

where $y_{iat}$ is the health outcome and $x_{it}$ the exposure of interest measured at time *t*. The term *g* represents a link function, while $\alpha_{ia}$ is the model intercept. The most common design for this first-stage analysis is time series,^20^ in which case $y_{iat}$ is a (quasi-)Poisson count variable and *g* is the log-link. Other designs such as the case-crossover^27^ or case time-series^28^ can easily be applied, in which case $y_{iat}$ can either be Binary or Poisson,^29^ and $\alpha_{ia}$ will be defined by strata.

The association of interest is represented by the flexible function *f* that can be non-linear and includes lags *l*. This function is uniquely defined by the parameter vector $\theta_{ia}$ , and it can represent associations of various complexity from a simple linear form to a complex cross-basis for distributed lag non-linear models (DLNM).^30^ The functions $s_{j}$ and $h_{p}$ , with corresponding vector coefficients $\varphi_{iaj}$ and $\gamma_{iap}$ , represent additional terms used to control for time trends and measurable time-varying confounders $z_{iapt}$ (potentially age-specific), respectively. The former are functions of time and can involve time-related variables such as day of week year to model variations in the long, seasonal, or weekly timescales.

#### 3.1.2 Application

The present application follows a time series design, in which $y_{iat}$ and $x_{it}$ are mortality counts and mean temperature series, respectively, and model (1) is a quasi-Poisson regression. *f* specifies a standard DLNM to model a non-linear association between $y_{iat}$ and $x_{it}$ .^30^ DLNM specification and parametrisation are discussed in previous research,^3,30,31^ but briefly it includes a B-spline of degree 2 with knots at the 10^th^, 75^th^ and 90^th^ percentiles of $x_{it}$ on the exposure dimension, and a natural spline of lags up to 21 days with three knots at equally-spaced values on the log-scale. The $s_{j}$ terms include a natural spline of time with 7 degrees of freedom per year, as well as day-of-week indicators. No measurable time-varying confounder $z_{iapt}$ is considered in this example. We additionally reduce the parameter vector by cumulating it along the lag dimension to obtain a lower dimension vector $\theta_{ia}$ .^32^ This step then results in a set of 300 (five age group within 60 observed cities) estimated vectors of 5 parameters ${\hat{\theta }}_{ia}$ along with their variance–covariance matrices $S_{ia}$ .

### 3.2 Modelling demographic differences

#### 3.2.1 Motivation and description

When age groups *a* are defined in Section 3.1, the next step is to attribute an age variable $A_{ia}$ to each estimate ${\hat{\theta }}_{ia}$ . When age groups are common to all cities, we can simply consider a factor variables $A_{ia}=a$ . However, when age groups are inconsistent, or otherwise to allow disaggregation of the risks, it is possible to construct a continuous meta-variable $A_{ia}$ representing the average age of death within each age group for city *i*. The simplest definition of $A_{ia}$ would be the middle point of each age group, for example $A_{ia}=70$ for the 65–74 age group. When information about age-specific deaths is available, this variable can be improved to better represent the distribution of deaths within an age group, by attributing more weight to ages with higher death rates, that is

(2) $$A_{ia}={\left(\overset{u}{\underset{k=l}{\sum }}\;d_{ik}\right)}^{-1}\overset{u}{\underset{k=l}{\sum }}\;o_{ik}d_{ik}$$

with $o_{ik}$ the age and $d_{ik}$ number of deaths for age *k* in location *i*. Here, *l* and *u* represent the lower and upper bound of age group *a* in location *i*, respectively. When *u* is not defined, $A_{ia}$ can be set to the life expectancy at age *l* for location *i*. If life expectancy information is not available, an arbitrary upper bound can be set such as u=100 . Computing $A_{ia}$ such as in equation (2) allows to better represent the local distribution of deaths which can differ from city to city, especially for wide age groups such as 0–65. The meta-variable $A_{ia}$ can then be included as a predictor in the second-stage meta-analysis to model age differentials in a flexible, continuous manner.

#### 3.2.2 Application

In the present application, we apply formula (2) for age groups 0–44, 45–64, 65–74 and 75–84, and life expectancy at 85 for age group 85+. For the youngest age group, this results in values of $A_{ia}$ between 24.7 in Modena and 29.2 in Trieste, illustrating different age structures of deaths.

### 3.3 Composite indices of vulnerability

#### 3.3.1 Motivation and description

Vulnerability to an environmental stressor depends on many local characteristics that can feature the local climate as well as socio-economic, topologic and environmental conditions.^33^ These factors are therefore predictors of the local risk represented by the vector $\theta_{ia}$ . However, there can be many potential predictors, and several of them are usually highly correlated. This makes the inclusion of these drivers in the second-stage meta-analytical model (see Section 3.4) computationally challenging, especially in an already complex multivariate/multilevel modelling context. In fact, previous studies included only a limited number of pre-selected meta-predictors.^34–36^

To combine the contributions of many potential drivers of vulnerability, we resort to dimension-reduction techniques. Using a (potentially large) number *P* of local characteristics for location *i*, defined by the P-length vector $v_{i}$ , dimension reduction seeks to create a limited number K≪P of components $w_{i}=R^{\text{T}}v_{i}$ as linear combinations of original variables, with R as a matrix of loadings defining orthogonal rotations. Such transformed variables $w_{i}$ are constructed to allow the first components to include a large part of the original information in $v_{i}$ . The most common example of dimension-reduction methods is principal component analysis (PCA), in which R is estimated to maximise the variance information contained in $w_{i}$ while ensuring the derived variables are uncorrelated. However, there are alternatives such as canonical correlation analysis (CCA), which creates new components $w_{i}$ to maximise their correlation with an outcome (here ${\hat{\theta }}_{ia}$ ), or partial least-squares (PLS), which maximises both the variance contained in $w_{i}$ and its correlation with an outcome.^37^

#### 3.3.2 Application

**Figure 2.**
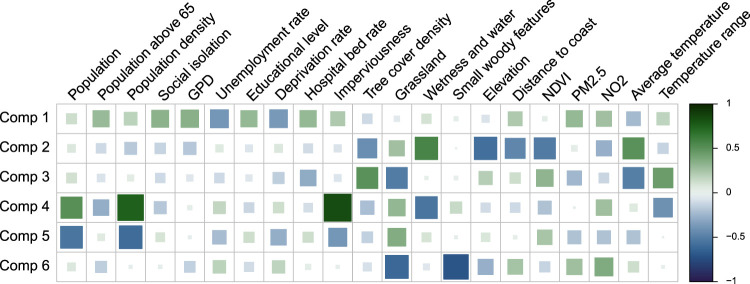
Linear correlation between meta-predictors and partial least-squares (PLS) components. Square size and colour both indicate the correlation magnitude. For color figure, see online version.

We choose PLS as it tends to more efficiently summarise the information in a limited number of components, especially when the objective is then to predict an outcome.^37^ To illustrate the nature of these composite indices of vulnerability, Figure 2 shows the correlation between the first six PLS components obtained on the Italian dataset and the original 21 city-specific characteristics. While the interpretation of each component is somewhat subjective, the plot shows for instance that the second component tends to represent greener and inland cities, while component 4 represents an urban-rural separation.

### 3.4 Predictive meta-regression model

#### 3.4.1 Motivation and description

Once first-stage coefficients representing city and age group-specific ERFs ${\hat{\theta }}_{ia}$ together with age $A_{ia}$ and location-specific composite vulnerability indices $w_{i}$ have been derived, they can be linked in a second-stage meta-regression model. This model addresses two objectives: (i) the provision of average ERFs and impact measures at different geographical levels such as regions or countries; and (ii) predict exposure-response functions (ERF) at locations with no health outcome data but with city-specific characteristics available.

The predictive model can be expressed as a multilevel multivariate meta-regression model,^14^ defined as:

(3) $${\hat{\theta }}_{ia}=X_{ia}\beta +Z_{i}b_{i}+\epsilon_{ia}$$

where ${\hat{\theta }}_{ia}$ are the estimated parameters representing city and age-group specific exposure-response relationships from the first stage, β is a fixed effect coefficient vector associated to the design matrix $X_{ia}$ , $b_{i}$ a city-specific random effect coefficient vector with design matrix $Z_{i}$ and $\epsilon_{ia}$ is the residual capturing uncertainty of first-stage estimates. Both $b_{i}$ and $\epsilon_{ia}$ are centred Gaussian vectors, with respective variance–covariance matrices $\Psi_{\text{i}}$ and $S_{ia}$ . Model (3) is flexible and encompasses many common meta-analysis methods encountered in the literature including fixed and random-effect meta-analysis, as well as multilevel meta-regression.^15^ Model (3) allows propagating uncertainty from the first-stage estimates ${\hat{\theta }}_{ia}$ , by attributing higher weights to observations with low variance covariance matrices $S_{ia}$ in the fitting of the model.

From model (3), best linear unbiased predictions (BLUPs) are usually computed, in which locations with high first-stage variance are pulled towards locations with more accurate first-stage estimate and similar fixed-effect values, that is

(4) $${\hat{\theta }}_{ia}^{b}=X_{ia}\hat{\beta }+Z_{i}{\hat{\Phi }}_{i}Z_{i}^{T}\Sigma_{ia}^{-1}({\hat{\theta }}_{ia}-X_{ia}\hat{\beta })$$

where $\Sigma_{ia}=S_{ia}+Z_{i}\Psi_{i}Z_{i}^{T}$ is the marginal variance of the ${\hat{\theta }}_{ia}$ vector. In unobserved locations ${\hat{\theta }}_{ia}$ and $\Sigma_{ia}$ are unavailable and the BLUP (4) cannot be computed. In these locations, predictions of exposure-response coefficients are then limited to fixed effects, that is ${\hat{\theta }}_{ia}^{f}=X_{ia}\hat{\beta }$ . This prediction can be performed for both observed and unobserved cities and age groups.

#### 3.4.2 Application

In the illustrative example, the fixed-effect matrix $X_{ia}$ contains the age variable $A_{ia}$ , expanded through natural splines with a single knot at age 60 to represent a non-linear age effect, as well as the first six composite indices of vulnerability in $w_{i}$ described in Section 3.3. The spline specification of $A_{ia}$ and the number of composite components were chosen by comparing the Akaike Information Criterion (AIC) of different models, as described in supplementary materials.^14,38^ The term $Z_{i}b_{i}$ is specified as a city-level random intercept (with $Z_{i}=I_{5}$ ) to further capture city-specific differences that are not linked to any factors considered in the prediction. We fit model (3) by restricted maximum likelihood (REML) as generally recommended.^14^

**Figure 3.**
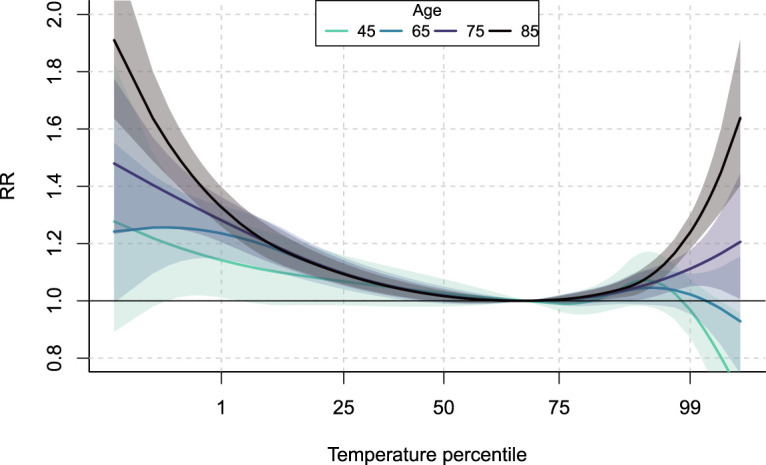
Average exposure-response relationships at various age values predicted from the meta-regression model. Shaded areas indicate the 95% confidence interval. For color figure, see online version.

**Figure 4.**
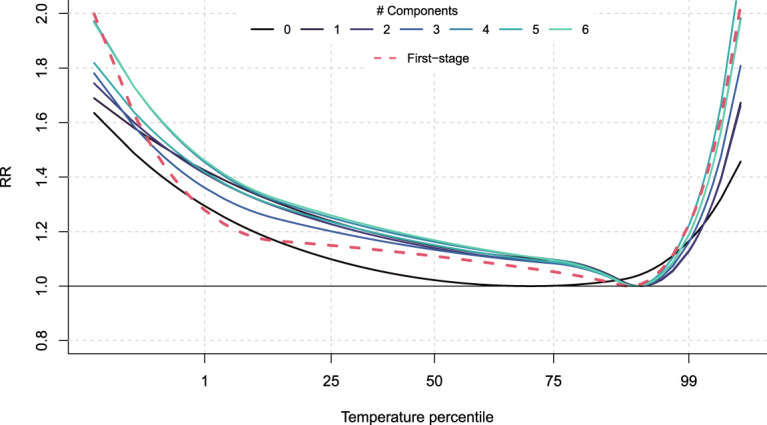
Predicted overall cumulative exposure-response relationships for the age group 75–85 in the city of Turin when including different number of PLS components. For color figure, see online version.

To illustrate disaggregation of age groups, Figure 3 shows the average ERF predicted at four different age values when composite indices $w_{i}$ correspond to average levels of city-specific characteristics $v_{i}$ . The comparison of the curves indicates that age a is a major effect modifier with the risk of both heat and cold being lower at age 45 compared to older ages such as 85, consistently with the literature.^22,24,39^ The increase of risk with age is steeper for heat, while for cold the differences are more noticeable for extreme temperatures. To demonstrate the role of city-level characteristics in capturing differential risk patterns, Figure 4 shows an example of predicted ERF for the city of Turin with an increasing number of composite indices of vulnerability in the predictive model. It shows that considering more components, and thus more information from vulnerability factors, allows predicted ERF to match more closely the first-stage estimated one. This is especially true for the heat part of the curves, which gets closer to the first-stage estimate as the number of indices increases, reaching an almost perfect match with the selected six indices.

### 3.5 Spatialisation of risk

#### 3.5.1 Motivation and description

Despite the wealth of information included in the predictive model of equation (3), fixed effect predictions ${\hat{\theta }}_{ia}^{f}$ could be insufficient to capture spatial patterns, as illustrated on the cold part of the curves in Figure 4. These unexplained patterns can come from missing key variables or misspecification of the mixed-effects meta-regression model and are usually partly captured by BLUP residuals ${\hat{\xi }}_{i}={\hat{\theta }}_{ia}^{b}-{\hat{\theta }}_{ia}^{f}=Z_{i}{\hat{\Phi }}_{i}Z_{i}^{T}\Sigma_{ia}^{-1}({\hat{\theta }}_{ia}-X_{ia}\hat{\beta })$ . BLUP residuals correspond to the difference between fixed-effect prediction ${\hat{\theta }}_{ia}^{f}=X_{ia}\hat{\beta }$ and the full BLUPs ${\hat{\theta }}_{ia}^{b}$ (4).

BLUPs residual coefficients ${\hat{\xi }}_{i}$ are only defined for observed locations. The objective of this step of the analysis is therefore to extrapolate the BLUP residuals ${\hat{\xi }}_{i}*$ at unobserved locations from observed neighbours through geostatistical methods. A large variety of methods are available to perform such extrapolation, such as Thiessen polygons, inverse distance weighting,^40^ as well as methods based on continuous Gaussian random fields such as kriging^41^ or the integrated nested Laplace approximation (INLA).^42^

#### 3.5.2 Application

**Figure 5.**
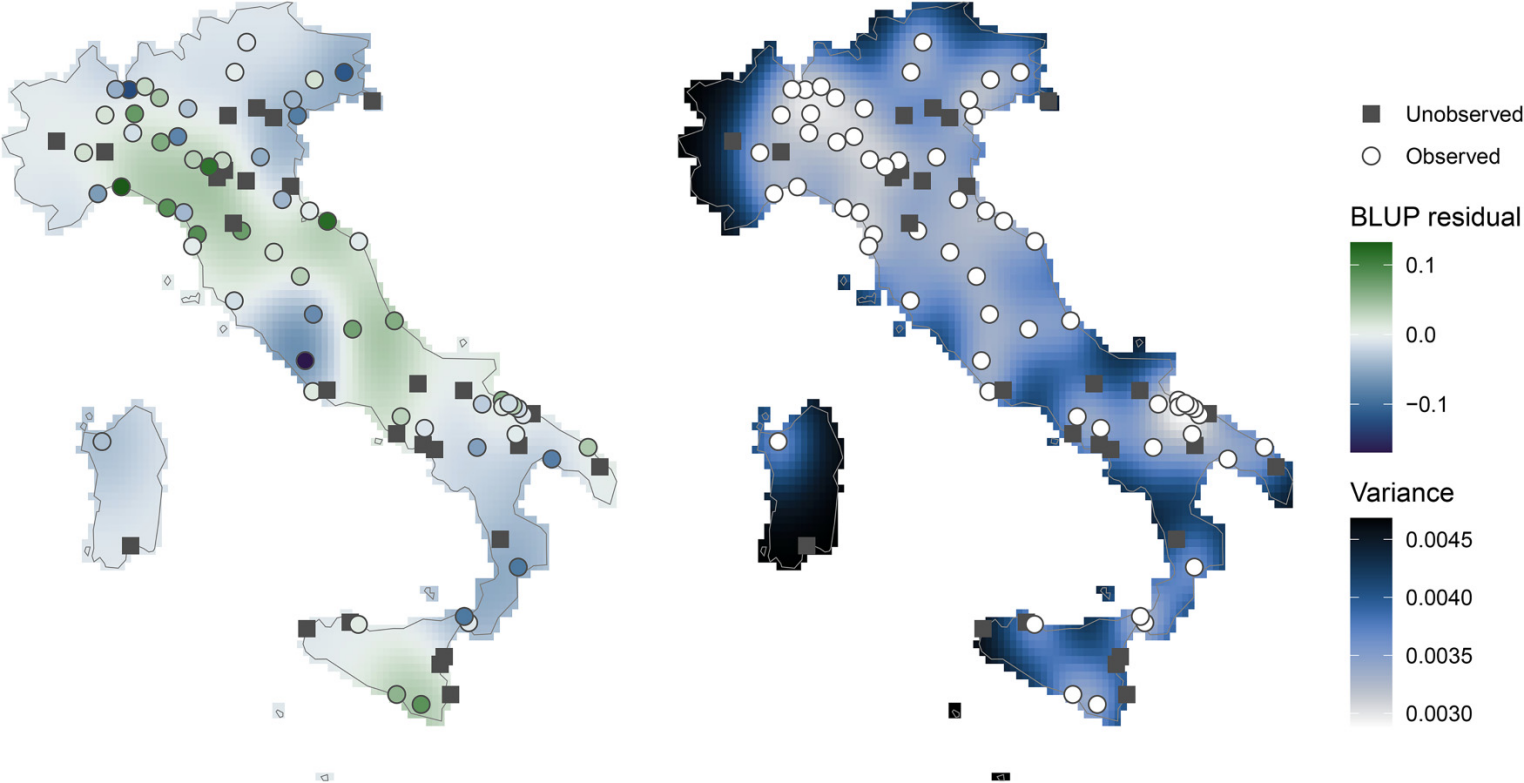
Maps of BLUP residuals of the second spline coefficient ${\hat{\xi }}_{i1}$ obtained by kriging, with predicted residual on the left-hand and associated variance in the right-hand panels. For color figure, see online version.

In this application, we consider kriging, which is a well-established geostatistical method that results in the BLUP in the context of Gaussian random fields, while being easily extendable to multivariate outcomes and offering measures of variance of the extrapolation. More specifically, an isotropic kriging is performed considering a Gaussian model with nugget effect fitted by least-squares on the empirical semi-variogram. We describe kriging in more detail in supplementary materials. In our illustrative example, the map in the left-hand panel of Figure 5 shows BLUP residuals ${\hat{\xi }}_{i1}$ for observed cities corresponding to the first spline coefficient. In the B-spline parametrisation, this coefficient can be loosely related to the extreme cold part of the curve and corresponds to a temperature range with important discrepancies between the first stage and prediction, as illustrated in Figure 4. The same map also shows the kriged surface of ${\hat{\xi }}_{i1}*$ , indicating patterns with positive residuals in the central regions (except near Rome) and negative residuals in the Alpine and Southern areas. The map in the right-hand panel of Figure 5 shows the variance of the prediction ${\hat{\xi }}_{i1}*$ , which as expected is higher in remote locations with respect to the observed cities. For instance, the predicted BLUP residual in Cagliari (south of Sardinia) is less accurate than in the well-covered North of Italy.

### 3.6 Prediction to unobserved locations

#### 3.6.1 Motivation and description

Once the predictive meta-regression (3) and the geo-spatial models are fitted, the risk associated with the exposure of interest can then be fully predicted for unobserved locations. We apply the following prediction process: (i) compute the composite indices of vulnerability $w_{i}*\;$ as in Section 3.3 using the known city-specific characteristics, and keep only the first components selected in the predictive model, (ii) using these indices, pre-specified age variable transformations and other potential variables included in the predictive meta-regression model, estimate the fixed-effect part of the spline coefficients ${\hat{\theta }}_{ia}^{f*}$ at the unobserved locations, (iii) estimate the random part ${\hat{\xi }}_{i}*$ from the kriging model fitted in Section 3.5, (iv) estimate coefficients by summing the fixed and random-effect part of the predicted coefficients ${\hat{\theta }}_{ia}^{b*}={\hat{\theta }}_{ia}^{f*}+{\hat{\xi }}_{i}*$ and (v) compute the variance–covariance matrix of the predictions by adding the variance–covariance matrices of ${\hat{\theta }}_{ia}^{f*}$ and ${\hat{\xi }}_{i}*$ . The variance–covariance matrices can be simply summed because fixed and random effects are assumed to be independent in the mixed-effect meta-analysis framework.^14^

#### 3.6.2 Application

**Figure 6.**
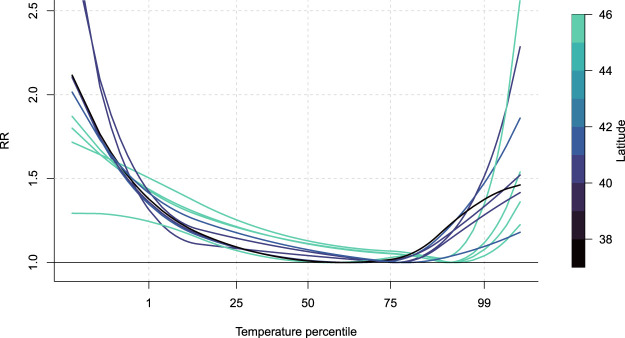
Predicted response functions at a sample of ten unobserved locations for age group 75–85. Colour gradient indicates latitude of the city, with darker colours indicating southern cities and lighter indicating northern cities. For color figure, see online version.

Examples of predicted curves are displayed in Figure 6 for age group 75–84 in a sample of ten unobserved cities. The plot shows how the framework allows to predict a wide range of ERF shapes and magnitude. The comparison of the curves highlights the discrepancy between northern and southern cities, even though latitude is not included in the meta-analytical model. Specifically, northern cities tend to have less steep relative risk curves for both heat and cold, which could be linked to less harsh summers and generally wealthier populations, as well as a much higher minimum mortality percentile, indicative of acclimatisation.

### 3.7 Age standardisation of impacts

#### 3.7.1 Motivation and description

Once predictions of the coefficients ${\hat{\theta }}_{ia}^{b*}$ characterising ERFs are obtained from the modelling described above, we can assess the impact of the exposure on the target population. For a specific exposition *x* and location *i* and group *a*, impacts are usually reported as attributable fraction ${\text{AF}}_{ia}^{x}=1-exp(-f(x,l;\;{\hat{\theta }}_{ia}^{b*}))$ and number ${\text{AN}}_{ia}^{x}={\text{AF}}_{ia}^{x}d_{ia}$ , where $d_{ia}$ is the total number of deaths. AF and AN quantify the excess mortality that can be attributed to the exposure in relative and absolute terms, respectively.^43^ For time series data, ${\text{AN}}_{ia}^{x}$ can be computed daily and summed over the whole study period to obtain an overall $\text{A}{\text{N}}_{ia}$ , a procedure that allows accounting for seasonality and long-term trends of deaths in estimating the public health impact. When series of mortality are unavailable, annual mortality can be rescaled using a day-of-year average in observed locations to emulate a seasonality effect.^44^

In large multi-location settings, these indices have important limitations in quantifying and comparing impacts of the exposure across populations.^45^ First, attributable measures offer an incomplete picture of the actual impact on a population, depending on its size and its outcome-specific mortality rate. Second, and more importantly, they do not account for demographic differences. Since both the age structure and age-specific risks usually vary between locations, the comparison of attributable numbers and fractions can be biased by demographic differences, and mask variation in vulnerability due to other factors, such as the environment or the socio-economic conditions of the populations. A more appropriate impact measure is the excess rate, which can be reported as both age-specific or standardised using a reference population.

Age group-specific excess mortality rates $E_{ia}*$ are computed by dividing the attributable number by the population, that is $E_{ia}*=\text{A}{\text{N}}_{ia}/p_{ia}$ where $p_{ia}$ is the population of age group *a* and location *i* over the study period. The standardised excess rate $E_{i}*$ in population *i* can then be computed by a weighted mean of age group-specific excess mortality rates $E_{ia}*$ as:

(5) $$E_{i}*={\left(\sum w_{a}\right)}^{-1}\underset{a}{\sum }\;E_{ia}*w_{a}$$

where weight $w_{a}$ is the age group-specific proportion or size of the reference population. The choice of the reference population for the computation of standardised rates is specific to the study settings and aims.

#### 3.7.2 Application

**Figure 7.**
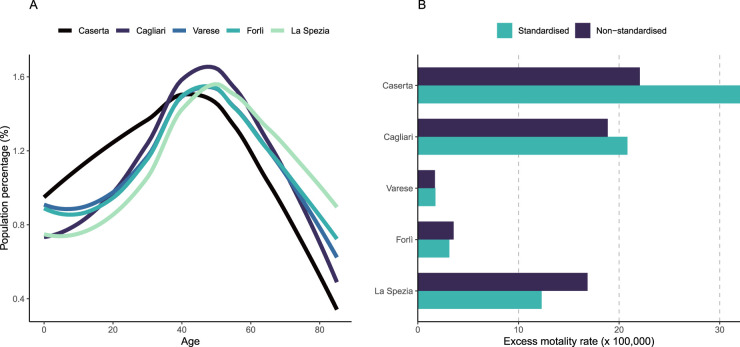
Standardisation of excess mortality rates in five Italian cities: (a) age distributions and (b) difference between standardised and non-standardised excess mortality rates. For color figure, see online version.

In this application, standardised rates (5) are computed using the average Italian demographic structure as a reference. Figure 7 illustrates the effect of standardisation on the excess mortality in five cities with different age structure. The extremes are Caserta, which displays a young population with a large proportion below 40 years old, and La Spezia, characterised by a much older population (left-hand panel). The right-hand panel shows that standardisation increases the estimated excess mortality rates in the younger cities of Caserta and Cagliari and decreases it in the older city of La Spezia. On the other hand, the figure is almost unchanged in Varese and Forli where the age structures are very close to the average Italian age structure.

### 3.8 Uncertainty assessment

#### 3.8.1 Motivation and description

Given the complexity of public health impact measures such as the AF or standardised excess mortality rates described above, closed-form formulae are not easily derived to define standard errors and confidence intervals for these measures.^46^ Therefore, we usually resort to empirical confidence intervals (eCI),^47^ computed by simulating a large number *N* of synthetic spline coefficients from city and age group-specific coefficients ${\hat{\theta }}_{ia}^{b*}$ predicted in Section 3.6. From the *N* simulated sets, we can then compute *N* measures of impacts $E_{i}*$ , and estimate confidence intervals by extracting the empirical quantiles from the *N* measures computed.

The established method to simulate synthetic spline coefficients is to sample from the central estimate ${\hat{\theta }}_{ia}^{b*}$ and the corresponding variance–covariance matrix $V({\hat{\theta }}_{ia}^{b*})$ . However, this approach ignores the dependency existing between location-specific predictions, inheriting from the fact that ${\hat{\theta }}_{ia}^{b*}$ all share the same fixed-effect part from the predictive meta-regression model. The consequence is that when aggregating measures of impacts, for example to summarise at the country or regional level, the derived eCIs tend to underestimate the actual uncertainty. This underestimation is especially visible when aggregating a large number of homogeneous locations, for instance with neighbourhoods of the same city.^24^

To account for this dependency, simulations must be performed from the original source of uncertainty, which is the predictive meta-regression model. Therefore, instead of simulating from predicted coefficients ${\hat{\theta }}_{ia}^{b*}$ , we simulate directly from the fixed-effect coefficients $\beta ∼N(\hat{\beta },V_{\beta })$ , from which we can then make predictions of ${\hat{\theta }}_{ia}^{f*}\;$ for each location and age group. To account for uncertainty in the spatialisation of random effects, we also simulate from the extrapolated BLUP residuals ${\hat{\xi }}_{i}*$ using $V({\hat{\xi }}_{i}*)$ , still assuming a normal distribution. Note that such simulations are not possible when extrapolation is made by Thiessen polygons or IDW, due to a lack of variance of estimates. The separate simulation of fixed and random parts of the BLUP is made possible by the assumption of independence between fixed and random effects in the meta-analysis framework.^14^ From this, we can then obtain synthetic predictions $\theta_{ia}^{b*}=\theta_{ia}^{f*}+{\hat{\xi }}_{i}*$ and proceed to the computation of impact measures and eCIs. Specifically, the aggregation must be applied separately to the output of each Monte Carlo iteration, and the empirical quantiles computed on the aggregated results, thus accounting for the correlations across locations.

#### 3.8.2 Application

**Figure 8.**
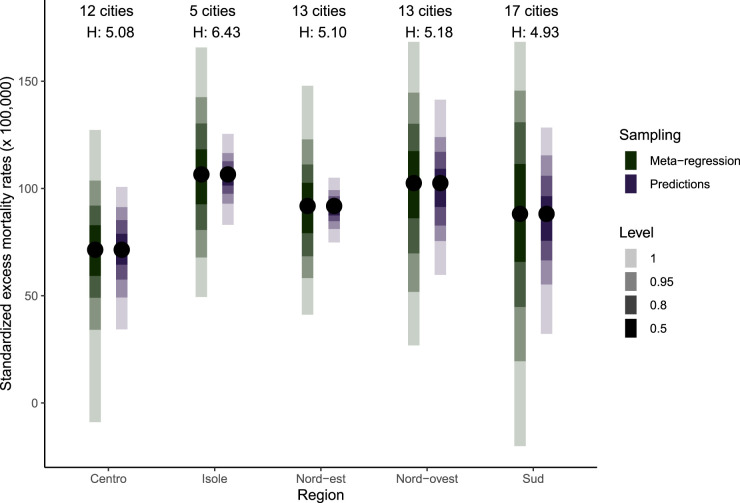
Distribution of standardised excess mortality rates over 1000 Monte Carlo simulations with the point estimate and 95% confidence intervals. Meta-regression indicates that simulations are performed at the meta-coefficients levels and predictions that simulations are performed using the predicted coefficients for each location and age group. The *H* score represents the regional heterogeneity statistic. For color figure, see online version.

Figure 8 shows various confidence intervals obtained through N=1000 simulations. Here, standardised excess death rates are shown, aggregated by five Italian administrative regions, each including between 11 and 26 cities. Figure 8 also indicates the region specific *H* statistic from Higgins & Thompson.^48^ The eCIs are consistently wider when assessed with the newly proposed method, especially in the regions Nord-Est (North-East) and Isole (Islands), which contain smaller cities. These two regions show higher heterogeneity according to the *H* statistic than the Centro (Central) and Sud (South) regions. This shows that the underestimation of confidence intervals in using the old method increases with the heterogeneity of aggregated locations.

## 4 Validation study

We evaluate the predictive power of the proposed framework on the holdout cities selected in section 2. The reference to which the framework is compared is a classical two-stage analysis of the full set of cities. This is done by applying steps 1 to 4 of the predictive framework (first-stage to predictive meta-regression model), and computing predicted relationships for the 27 unobserved cities. All methodological details, including the specification of the DLNM cross-basis and the terms of the predictive meta-regression model, are identical to the application described in Section 3. We therefore compare the computed reference BLUPs to the predicted ERF when these 27 cities are considered unobserved.

**Figure 9.**
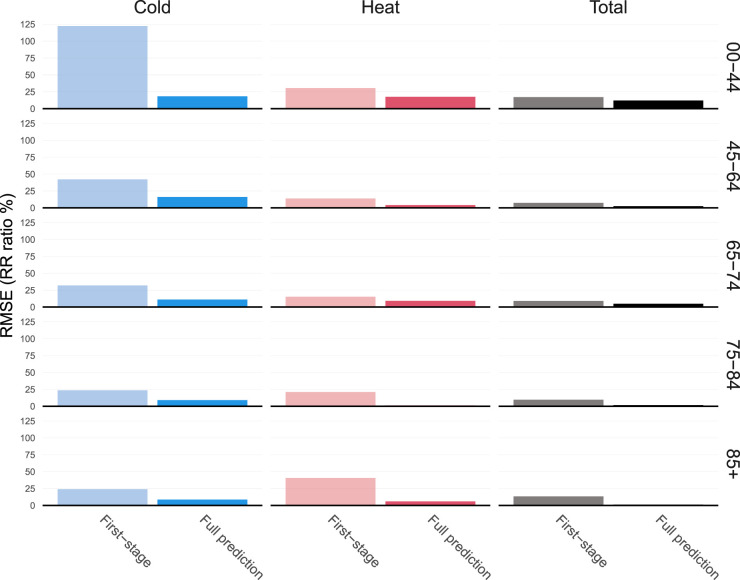
Root mean squared error (RMSE) of first-stage and predicted exposure-response functions (ERF) compared to best linear unbiased predictions (BLUPs) from a full two-stage analysis. RMSE is expressed as a relative increase on the relative risk (RR) scale, that is as exp(RMSE) – 1. The Total column represents RMSE computed on the whole ERF while the Cold and Heat columns restrict the RMSE on the temperature below the 5^th^ percentile, and above the 95^th^ percentile, respectively. For color figure, see online version.

Figure 9 shows the root-mean-squared error between predicted ERFs and the reference ERFs described above for the 27 unobserved cities, and compares it to the first-stage estimates for these same cities. It indicates the predicted ERFs tend to be close to the reference ones with a RMSE generally below 20%, and almost null for Heat and Total in the oldest age groups. This is confirmed by the predicted ERF in supplementary material that tend to be close to the BLUP ones. By contrast, there is a large difference between first-stage ERFs and the reference ones, showing that the prediction framework is able to capture differences between locations and age groups.

Comparing the different columns of Figure 9, it is clear that predictive performances are better in the middle of the ERF, and are usually the worst on the cold part of the ERF curve. There are also large differences between age groups, with the biggest RMSE found for the youngest group. These differences highlight poorer prediction performances where uncertainty is usually at its highest because of low mortality counts.

## 5 Discussion

In this contribution, we propose a cohesive set of modelling extensions for the two-stage framework used for risk estimation and health impact assessment in environmental epidemiology. This framework provides an accurate prediction of risks and impacts of environmental exposure on a health outcome, harnessing the modelling of major drivers of vulnerability, including age as well as any local characteristic potentially modifying the risk associated with the exposure. The inclusion of these local characteristics in a predictive meta-regression model, together with the spatialisation of the random part of BLUPs, provides an improved spatial representation for locations with no available outcome data. Finally, this framework offers improvements in the quantification of impacts, through the computation of standardised excess mortality rates to account for local age structures, and through a revised Monte Carlo simulation scheme that better represents the original source of uncertainty in the modelling.

This new framework builds upon previous work on two-stage modelling strategies, and it extends their applicability in a variety of situations including complex multilevel structures or longitudinal studies.^15^ The generation of flexible predictive models that incorporate contributions from various effect modifiers, as well as the separate modelling of fixed and random parts of BLUPs, the latter using geostatistical methods, extends the capabilities of multi-location studies. This has enabled large scale analysis of differential risks by age group,^49^ as well as substantially increasing the coverage of recent health impact assessments.^22^ This framework have also been used to downscale risks and impacts at small scales where power is lacking to obtain accurate estimates.^24^ This framework can be applied to study the health impacts in various scenarios, whether it is the implementation of urban policies aiming to increase the tree cover density,^50^ disentangling the role of specific components of air pollutants,^35^ or adaptation pathways in future climate.^51^

There are still important limitations to the applicability of this new framework. First and foremost, several stages rely on the availability of a consistent database of location-specific characteristics. This is not an issue in areas such as Europe or the United States, but it is an important limitation in areas with more data scarcity such as lower- and middle-income countries. Besides, the complexity of the framework necessitates a large number of available locations to achieve good predictive power. The validation study, along with previous studies including a multi-city analysis in Europe,^22^ show that there remain uncaptured risk differentials, even after the spatialisation of BLUP residuals. Indeed, contributions of vulnerability drivers can be highly complex, with potentially non-linear effect modifications and interactions between local characteristics. Modelling such complex patterns is possible in theory, although in practice it poses important methodological and computational problems. Finally, the framework is focused purely on risk prediction, with the intent to provide an accurate picture of risks and impacts of the exposure of interest. In particular, the use of dimension reduction techniques in the second stage is valuable for combining contribution of multiple vulnerability drivers, but it is not suitable to infer independent effect modification of individual local characteristics.

The validation study performed here shows satisfactory predictive performances by the proposed framework, but it remains to be evaluated at a larger scale. Indeed, Italy remains a populous country with clear heat-related mortality effects. Poorer performances on the youngest age group suggest that the predictive performances of the framework could be lower in more modest contexts. The context of multi-location studies in environmental epidemiology also renders any validation difficult, since there is no clear outcome variable to be predicted by the modelling. First-stage ERF estimates are subject to important uncertainties, and mortality time series depend on many location- and age-specific factors that need to be controlled for. Therefore, additional research is needed on the evaluation of such a framework to conduct health impact assessments in understudied locations such as the African continent or the Middle-East.

The limitations exposed above outline the need for further methodological development, specifically focusing on risk prediction in order to capture the high complexity of vulnerability to environmental exposures. This includes the development of machine learning methods for meta-regression to improve fixed effect prediction,^21^ as well as methods to integrate the spatialisation of risk, such as spatially structured random effects. Additional work is also needed on the evaluation of such a framework as the predictions are here compared to BLUPs which presents uncertainties and inherently depends on the chosen meta-regression model.

In conclusion, this contribution illustrates a modelling extensions for multi-location analyses of environmental risk factors, addressing known limitations of established methods. The framework builds on recent developments in two-stage designs and meta-analytical methods, as well as implementing sophisticated statistical techniques to model complex associations that vary between and within populations.

## Supplemental Material

sj-zip-1-smm-10.1177_09622802241313284 - Supplemental material for Modelling extensions for multi-location studies in environmental epidemiology Supplemental material, sj-zip-1-smm-10.1177_09622802241313284 for Modelling extensions for multi-location studies in environmental epidemiology by Pierre Masselot and Antonio Gasparrini in Statistical Methods in Medical Research

sj-docx-2-smm-10.1177_09622802241313284 - Supplemental material for Modelling extensions for multi-location studies in environmental epidemiology Supplemental material, sj-docx-2-smm-10.1177_09622802241313284 for Modelling extensions for multi-location studies in environmental epidemiology by Pierre Masselot and Antonio Gasparrini in Statistical Methods in Medical Research
