# Supplementary figures and images for: Modelling extensions for multi-location studies in environmental epidemiology
Source: Stat Methods Med Res. 2025 Feb 5;34(3):615–29. doi: 10.1177/09622802241313284 (PMC11951449; doi:10.1177/09622802241313284)

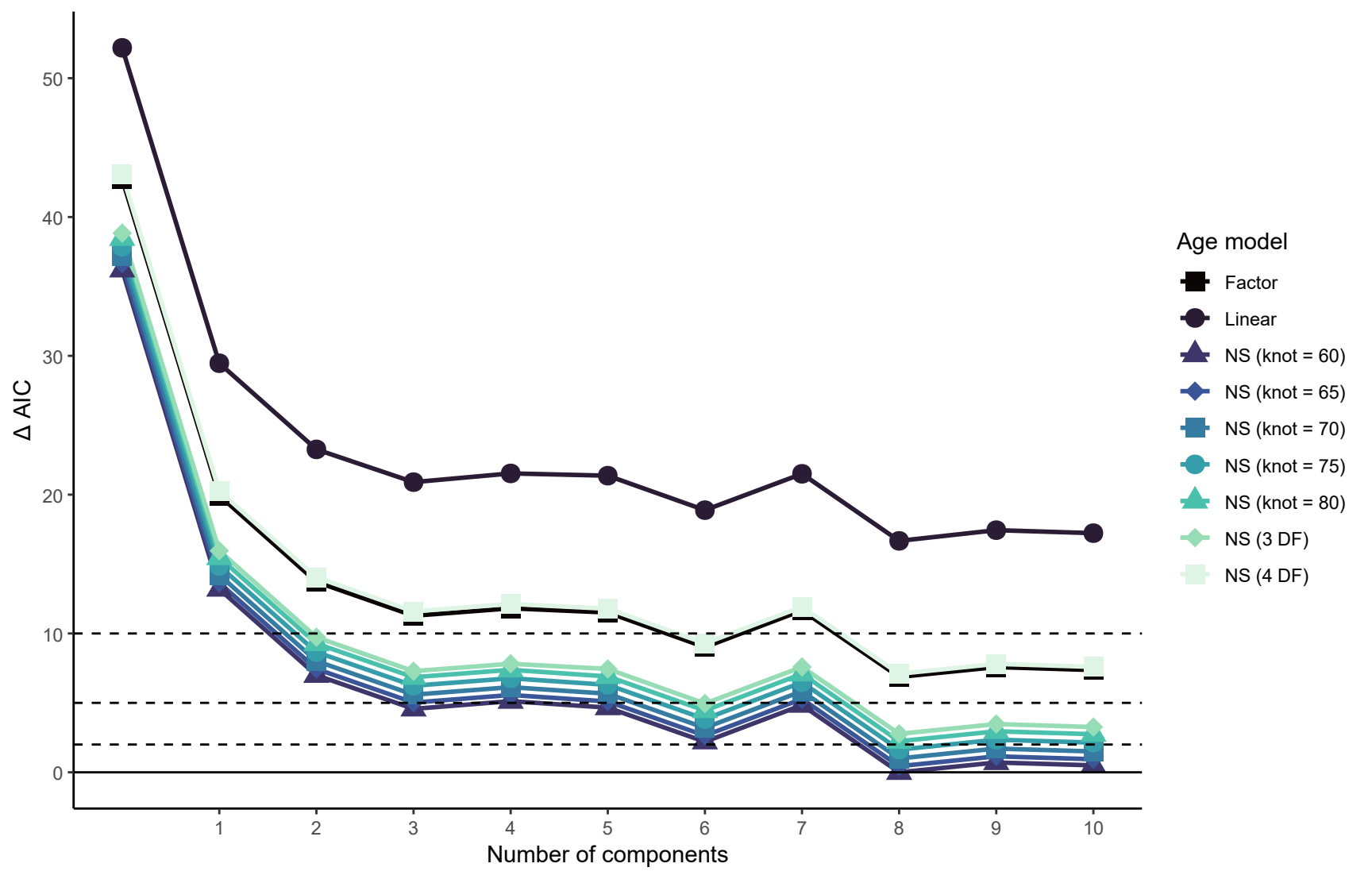

Supplement: sj-zip-1-smm-10.1177_09622802241313284 - Supplemental material for Modelling extensions for multi-location studies in environmental epidemiology [file sj-zip-1-smm-10.1177_09622802241313284.zip › RiskExtrapolation-main/figures/FigS1_npcSelect.pdf]

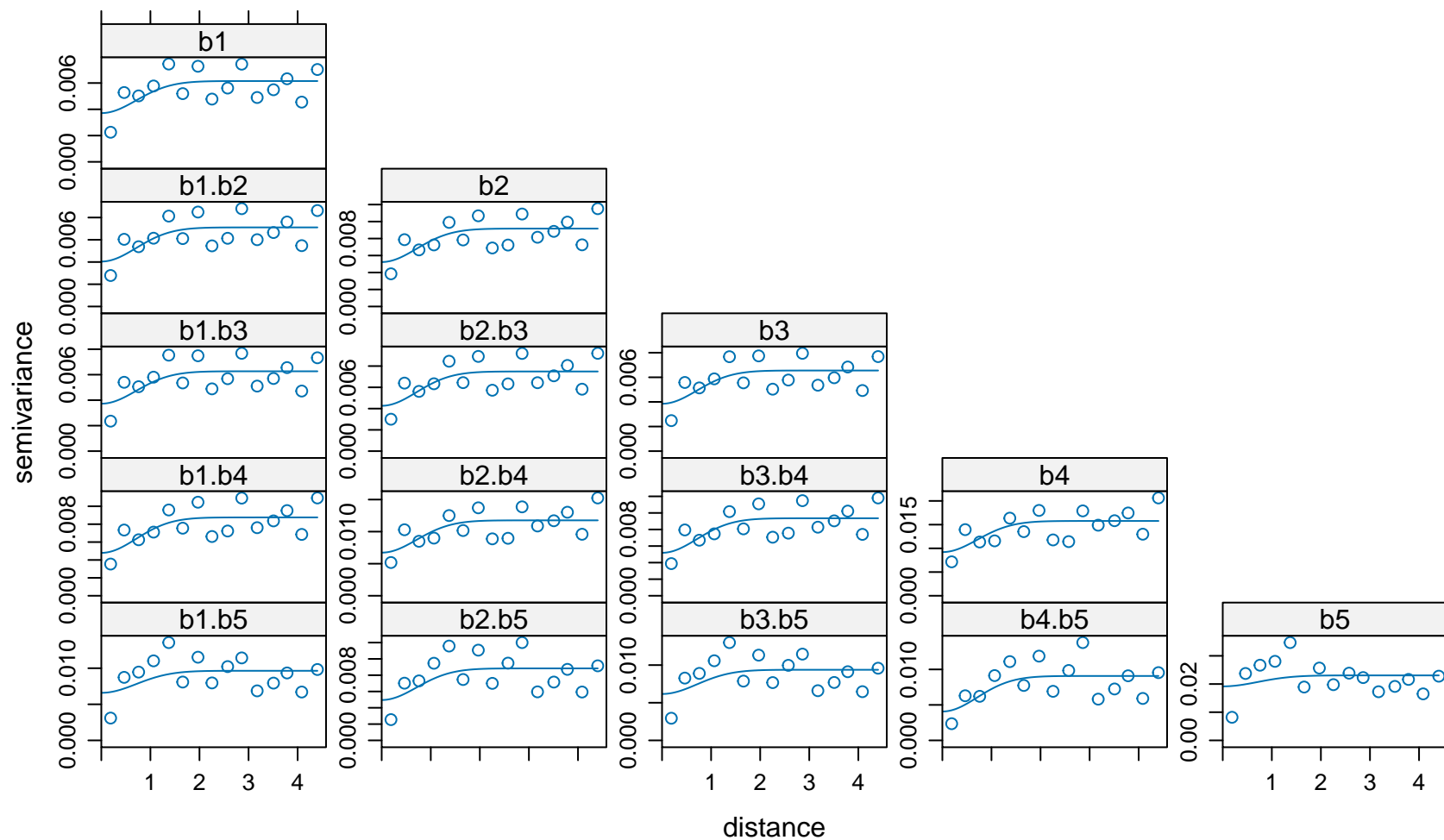

Supplement: sj-zip-1-smm-10.1177_09622802241313284 - Supplemental material for Modelling extensions for multi-location studies in environmental epidemiology [file sj-zip-1-smm-10.1177_09622802241313284.zip › RiskExtrapolation-main/figures/FigS2_variogram.pdf]
