# Supplementary material for: Modelling extensions for multi-location studies in environmental epidemiology
Source: Stat Methods Med Res. 2025 Feb 5;34(3):615–29. doi: 10.1177/09622802241313284 (PMC11951449; doi:10.1177/09622802241313284)
Supplement: sj-docx-2-smm-10.1177_09622802241313284 - Supplemental material for Modelling extensions for multi-location studies in environmental epidemiology [file sj-docx-2-smm-10.1177_09622802241313284.docx]

**A new modelling framework for multi-location studies in environmental epidemiology**

**Supplementary materials**

Pierre Masselot, Antonio Gasparrini

# Data description

The illustrative application includes 87 cities defined following Eurostat Urban Audit’s definition. The full list of cities is shown in Table S1. All metadata attached to cities are shown in Table S2.

Table S1: *List of cities considered in the illustrative example. Observed indicates whether the city is used to fit the second-stage meta-analysis model. Population and mean temperature are inherited from the parent study from which the metadata are extracted.*

| **Eurostat code** | **Name** | **Region** | **Observed** | **Population** | **Mean temperature** |
| --- | --- | --- | --- | --- | --- |
| IT001C | Rome | Centro | Yes | 2,672,838 | 15.8 |
| IT002C | Milan | Nord-ovest | Yes | 3,897,850 | 13 |
| IT003C | Naples | Sud | No | 3,047,442 | 16.2 |
| IT004C | Turin | Nord-ovest | No | 879,331 | 12.1 |
| IT005C | Palermo | Isole | No | 670,213 | 16.6 |
| IT006C | Genoa | Nord-ovest | Yes | 594,860 | 12.9 |
| IT007C | Florence | Centro | Yes | 365,265 | 13.7 |
| IT008C | Bari | Sud | No | 319,627 | 16.9 |
| IT009C | Bologna | Nord-est | No | 377,318 | 14.3 |
| IT010C | Catania | Isole | No | 305,233 | 18.2 |
| IT011C | Venise | Nord-est | Yes | 265,285 | 14.3 |
| IT012C | Verona | Nord-est | No | 256,724 | 13.5 |
| IT013C | Cremona | Nord-ovest | Yes | 71,222 | 14 |
| IT014C | Trento | Nord-est | Yes | 112,731 | 9.1 |
| IT015C | Trieste | Nord-est | No | 205,534 | 12.4 |
| IT016C | Perugia | Centro | Yes | 159,824 | 13.6 |
| IT017C | Ancona | Centro | Yes | 100,772 | 15.6 |
| IT018C | Pescara | Sud | Yes | 119,500 | 15.1 |
| IT019C | Campobasso | Sud | No | 49,892 | 13.1 |
| IT020C | Caserta | Sud | Yes | 76,201 | 15 |
| IT021C | Taranto | Sud | Yes | 199,740 | 17.4 |
| IT022C | Potenza | Sud | Yes | 67,678 | 11.8 |
| IT023C | Catanzaro | Sud | Yes | 91,832 | 16.2 |
| IT024C | Reggio Di Calabria | Sud | Yes | 181,257 | 15.1 |
| IT025C | Sassari | Isole | Yes | 124,625 | 16.8 |
| IT026C | Cagliari | Isole | No | 156,363 | 17.3 |
| IT027C | Padova | Nord-est | No | 208,056 | 13.9 |
| IT028C | Brescia | Nord-ovest | Yes | 191,362 | 12.7 |
| IT029C | Modena | Nord-est | No | 180,668 | 14 |
| IT030C | Foggia | Sud | No | 151,992 | 16.4 |
| IT031C | Salerno | Sud | No | 134,788 | 15.8 |
| IT032C | Piacenza | Nord-est | Yes | 101,375 | 13.8 |
| IT033C | Bolzano | Nord-est | Yes | 104,326 | 8.9 |
| IT034C | Udine | Nord-est | Yes | 98,925 | 12.2 |
| IT035C | La Spezia | Nord-ovest | Yes | 93,062 | 14.8 |
| IT036C | Lecce | Sud | No | 91,773 | 17.6 |
| IT037C | Barletta | Sud | Yes | 94,127 | 16.6 |
| IT038C | Pesaro | Centro | Yes | 94,580 | 15 |
| IT039C | Como | Nord-ovest | Yes | 83,189 | 11.8 |
| IT040C | Pisa | Centro | Yes | 87,945 | 15.5 |
| IT041C | Treviso | Nord-est | Yes | 82,689 | 13.3 |
| IT042C | Varese | Nord-ovest | Yes | 80,460 | 12 |
| IT043C | Asti | Nord-ovest | Yes | 74,811 | 13.1 |
| IT044C | Pavia | Nord-ovest | Yes | 70,380 | 13.9 |
| IT045C | Massa | Centro | Yes | 68,875 | 13 |
| IT046C | Cosenza | Sud | No | 68,131 | 14.3 |
| IT047C | Savona | Nord-ovest | Yes | 60,784 | 13.2 |
| IT048C | Matera | Sud | No | 60,026 | 15.6 |
| IT049C | Acireale | Isole | No | 51,768 | 16 |
| IT050C | Avellino | Sud | Yes | 54,493 | 13.2 |
| IT051C | Pordenone | Nord-est | Yes | 51,067 | 13.1 |
| IT052C | Lecco | Nord-ovest | Yes | 47,404 | 10 |
| IT053C | Altamura | Sud | Yes | 69,760 | 14.7 |
| IT054C | Bitonto | Sud | Yes | 55,456 | 16 |
| IT055C | Molfetta | Sud | Yes | 59,782 | 16.7 |
| IT056C | Battipaglia | Sud | No | 50,611 | 16.4 |
| IT057C | Bisceglie | Sud | Yes | 54,855 | 16.6 |
| IT058C | Carpi | Nord-est | Yes | 68,800 | 14.3 |
| IT059C | Cerignola | Sud | Yes | 57,146 | 16.3 |
| IT060C | Gela | Isole | Yes | 75,040 | 18 |
| IT061C | Bagheria | Isole | Yes | 54,402 | 17 |
| IT062C | Anzio | Centro | Yes | 51,135 | 17 |
| IT063C | Sassuolo | Nord-est | No | 40,495 | 13 |
| IT064C | Messina | Isole | Yes | 242,396 | 16.8 |
| IT065C | Prato | Centro | No | 185,458 | 13.4 |
| IT066C | Parma | Nord-est | Yes | 179,624 | 13.7 |
| IT067C | Livorno | Centro | Yes | 157,938 | 15.4 |
| IT068C | Reggio Nell'emilia | Nord-est | Yes | 160,771 | 13.5 |
| IT069C | Ravenna | Nord-est | No | 151,010 | 15 |
| IT070C | Ferrara | Nord-est | Yes | 132,369 | 14.7 |
| IT071C | Rimini | Nord-est | Yes | 139,996 | 14.9 |
| IT072C | Siracusa | Isole | No | 121,505 | 18.2 |
| IT073C | Bergamo | Nord-ovest | Yes | 116,270 | 11.7 |
| IT074C | Forlì | Nord-est | Yes | 114,818 | 14.2 |
| IT075C | Latina | Centro | No | 118,094 | 16.4 |
| IT076C | Vicenza | Nord-est | No | 111,772 | 13.1 |
| IT077C | Terni | Centro | Yes | 109,317 | 13.1 |
| IT078C | Novara | Nord-ovest | Yes | 102,591 | 13.2 |
| IT079C | Alessandria | Nord-ovest | No | 91,567 | 13.1 |
| IT080C | Arezzo | Centro | Yes | 98,352 | 13 |
| IT081C | Grosseto | Centro | Yes | 80,030 | 15.7 |
| IT082C | Brindisi | Sud | Yes | 87,851 | 17.5 |
| IT083C | Trapani | Isole | No | 68,439 | 17.7 |
| IT084C | Ragusa | Isole | Yes | 71,276 | 16.9 |
| IT085C | Andria | Sud | Yes | 99,678 | 15.2 |
| IT086C | Trani | Sud | Yes | 55,693 | 16.5 |
| IT087C | L'aquila | Sud | Yes | 69,158 | 9.2 |

Table S2: *List of city-level meta-variables. The first group represents the meta-predictors used in the second-stage meta-analysis, and the second represents variables used to compute impact measures.*

| **Variable** | **Source** | **Description** |
| --- | --- | --- |
| Total population | Urban Audit / Wikipedia | Note: population data were missing for several cities and were added from the Wikipedia pages for each city (Latvia: Valmiera; Belgium: Mechelen, Mouscron, La Louvière, Verviers) |
| Population above 65 | Urban Audit / NUTS3 | Percentage |
| Population density | Urban Audit / Wikipedia | Note: population density data were missing for several cities and were added from the Wikipedia pages for each city (Latvia: Valmiera; Belgium: Mechelen, Mouscron, La Louvière, Verviers; Ireland: Dublin, Limerick, Waterford; Norway: Bergen, Trondheim; UK: Glasgow, North Lanarkshire, Dundee) |
| Isolation | Urban Audit / NUTS2 | Proportion of single-person households |
| GDP | NUTS3 / Office of National Statistics | GDP per capita. Note: GDP data for UK cities were unavailable in Eurostat and extracted from ONS |
| Unemployment rate | NUTS2 | Among active population (20-64 years old) for all education levels |
| Education level | NUTS2 | Proportion of active population (25-64 years old) with ISCED level >= 5 (higher education) |
| Deprivation rate | NUTS2 | Proportion of population under severe material deprivation condition |
| Hospital bed rates | NUTS2 | Number of hospital beds / inhabitants |
| Imperviousness | Copernicus High Resolution Layer | Percentage of soil sealing |
| Tree Cover Density | Copernicus High Resolution Layer | Level of Tree Cover Density (%) |
| Grassland | Copernicus High Resolution Layer | Proportion of grassland pixels |
| Water & Wetness | Copernicus High Resolution Layer | Average class between (1) permanent water, (2) temporary water, (3) permanent wetness and (4) temporary wetness |
| Small Woody Features | Copernicus High Resolution Layer | Small woody features density (%) |
| Elevation | AWS Terrain Tiles | Elevation at city centre |
| Coastal region type | Natural Earth | Lowest distance between coastal line and city centre |
| NDVI | Google Earth Engine - MODIS |  |
| PM25 | Atmospheric Composition Analysis Group | https://sites.wustl.edu/acag/datasets/surface-pm2-5/ |
| NO2 | Atmospheric Composition Analysis Group | https://sites.wustl.edu/acag/datasets/surface-no2/ |
| Temperature range | Copernicus | Annual temperature range |
| Mean temperature | Copernicus | Mean annual temperature |
| Death rate | NUTS3 | Death rate within age group |
| Life expectancy | NUTS2 | Life expectancy at age |
| Population structure | NUTS3 | Proportion of the population in age group |

# Model selection

This section illustrates the model selection performed for the second-stage meta-analysis. Two parameters need to be chosen: i) the specification of the age variable, and ii) the number of composite components representing vulnerability. In a mixed-effect framework, model selection can be performed through the Akaike Information Criterion, following standard likelihood theory. Briefly, the (restricted) likelihood can be extracted from the estimated model, and the number of parameters is the sum of fixed effect coefficients $\boldsymbol{\beta}$ and parameters defining the random effect variance-covariance matrix $\boldsymbol{\Psi}_{i}$.^1^

Figure S1 shows the $\Delta AIC$, i.e. the difference in AIC with the overall minimum for various specifications. We choose the model with a natural spline and a single knot at age 60, and five composite indices, as the simplest model such that $\Delta AIC\leq2$, as recommended in the literature.^2^


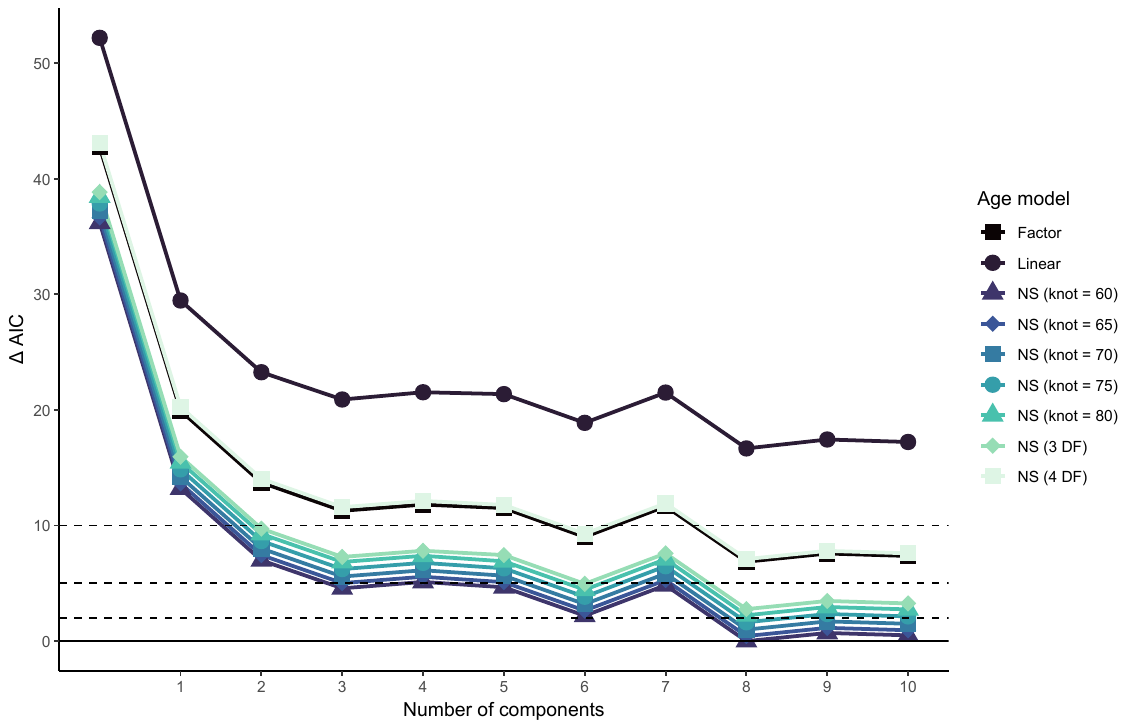


Figure S1: *Comparison of AIC for various number of composite indices and specification of the age term.* $\Delta AIC$*represents the difference between the model’s AIC and the minimum AIC from all models (represented by the thick horizontal black line). Horizontal dashed lines represent values of 2, 5 and 10 for* $\Delta AIC$.

# Description of Kriging

Kriging was introduced by Georges Matheron ^3^ in the context of mining, and has since been one of the most popular geostatistical methods, used in a wide variety of applications, including in environmental epidemiology ^4,5^. Using a set of $n$ realisations from a spatial process – in our case $\hat{\xi}_{i}=\hat{\xi}\left( s_{i} \right)$ where $s_{i}$ represents latitude and longitude – the objective of Kriging is to make a prediction of the process $\hat{\xi}\left( . \right)$ in a new location $s_{0}$. This prediction takes the form of a linear combination of the observed values:

|  | $\hat{\xi}\left( s_{0} \right)=\sum_{i=1}^{n} w_{i}\hat{\xi}\left( s_{i} \right)$ | (1) |
| --- | --- | --- |

with the weights $w_{i}$ summing to one. The best prediction, in the sense of minimising the prediction error variance $Var\left( \epsilon\left( s_{0} \right) \right)$, can be shown to be:

|  | $\boldsymbol{w}=[w_{1},\ldots,w_{n}]^{T}=\boldsymbol{C}_{n}^{-1}\boldsymbol{c}_{0}$ | (2) |
| --- | --- | --- |

where $\boldsymbol{C}_{n}$ is the covariance matrix of all observations $\hat{\xi}\left( s_{i} \right)$ and $\boldsymbol{c}_{0}$ is the vector of covariance between $\hat{\xi}\left( s_{0} \right)$ and all the observations $\hat{\xi}\left( s_{i} \right)$.

Equation (2) highlights the equivalence between Kriging and a classical linear regression with $\hat{\xi}\left( s_{0} \right)$ as the response variable and the $\hat{\xi}\left( s_{i} \right)$ as $n$ predictors. However, in contrast to a classical linear regression in which the covariance can be estimated by having several realisations for each variable ($\boldsymbol{C}_{n}=\boldsymbol{X}^{T}\boldsymbol{X}$ and $\boldsymbol{c}_{0}=\boldsymbol{X}^{T}\boldsymbol{y}$), in Kriging we dispose of only one observation. Therefore, we need to assume a parametric covariance function $C(d)$ with $d$ being the distance between two observations.

If the process $\hat{\xi}\left( . \right)$ is assumed to be stationary, the covariance function $C(d)$ is estimated from a variogram, a function giving the variance of the difference between two values $Var\left( \hat{\xi}\left( s_{i} \right)-\hat{\xi}\left( s_{j} \right) \right)$ according to their distance $d$. An empirical variogram $\hat{\gamma}\left( d \right)$can be estimated considering a step $\delta$ as

|  | $\hat{\gamma}\left( d \right)=\frac{1}{2N_{d}}\sum\left( \hat{\xi}\left( s_{i} \right)-\hat{\xi}\left( s_{j} \right) \right)^{2}$ | (3) |
| --- | --- | --- |

considering all pairs of observations $\hat{\xi}\left( s_{i} \right), \hat{\xi}\left( s_{j} \right)$ with a distance within $d\pm\delta$. From this empirical variogram, a variogram function $\gamma\left( d \right)$ following a predefined parametric shape can be fitted and then used to compute the covariances needed in Equation (2). For instance Figure S2 shows the variogram for each element of the BLUP residuals, with a fitted Gaussian model.


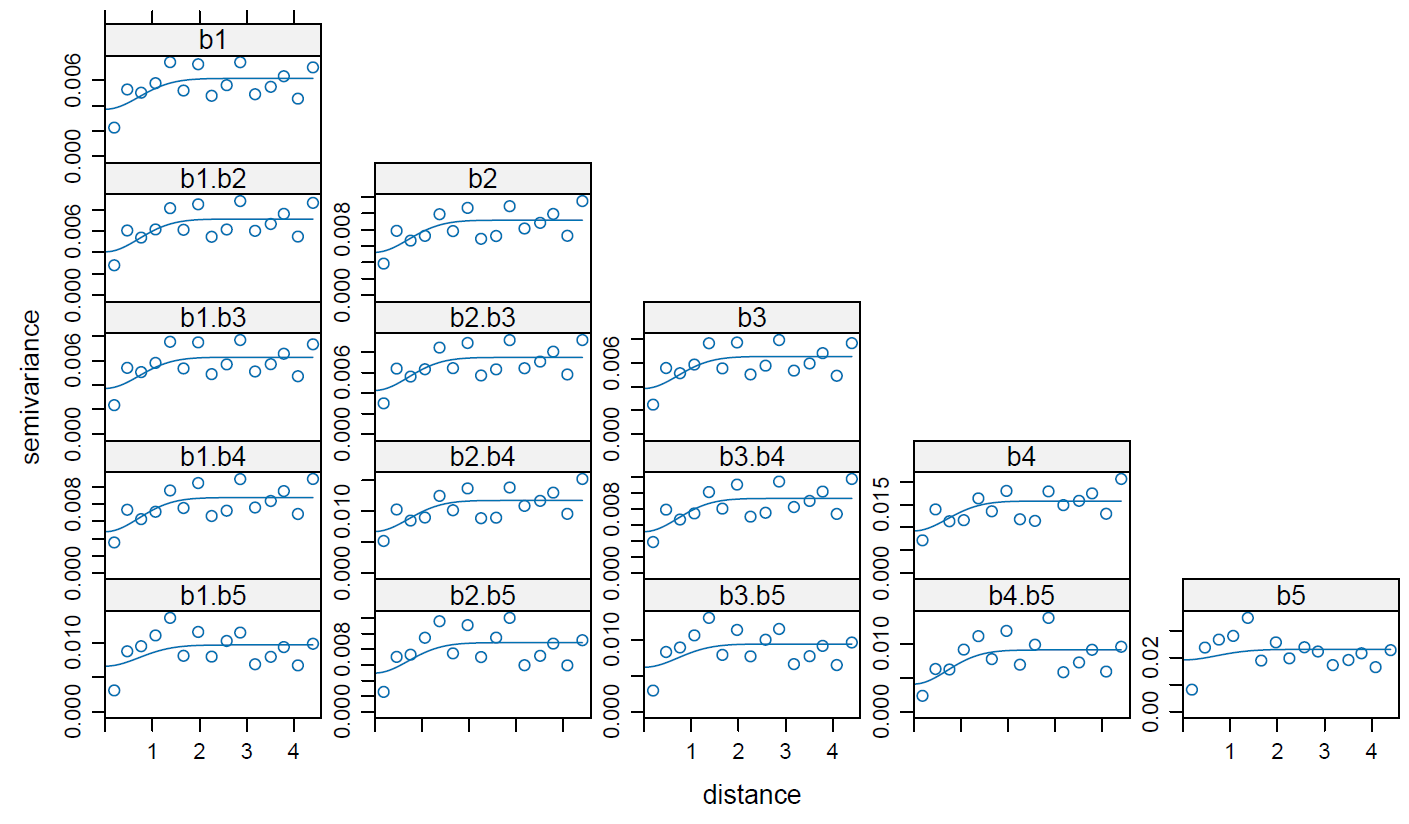


Figure S2: *Empirical variogram with fitted variogram model for all elements of the BLUP residuals* ${\hat{\boldsymbol{\xi}}}_{i}$*, including their covariance*.

# Additional validation results


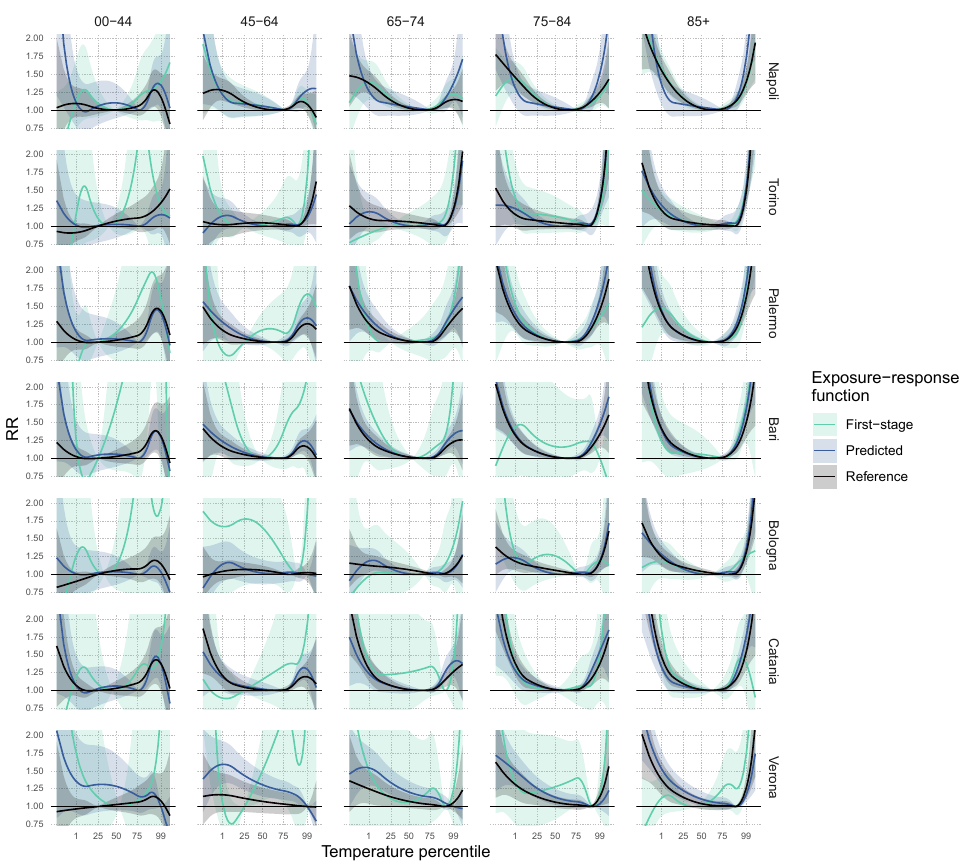


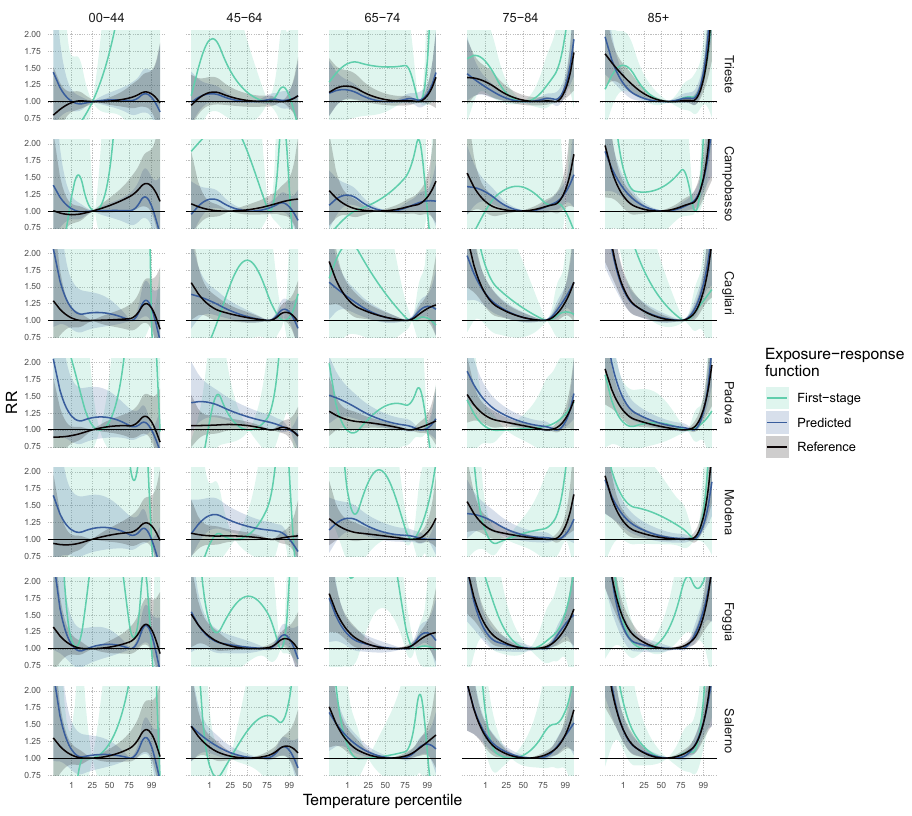


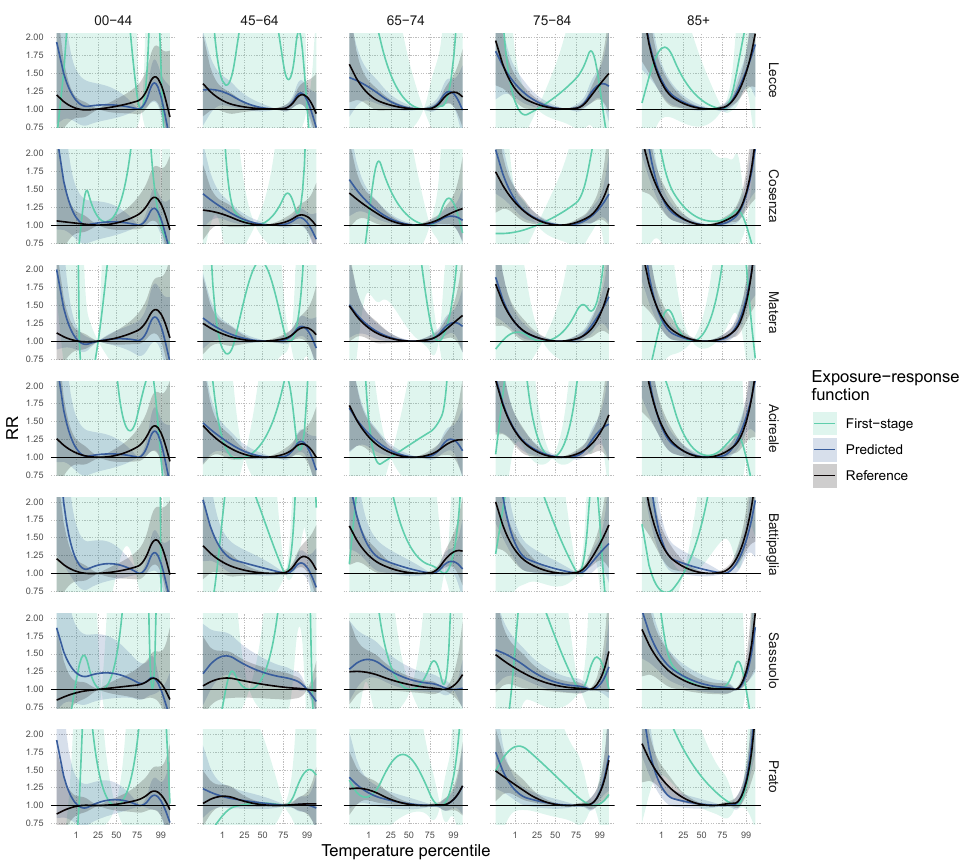


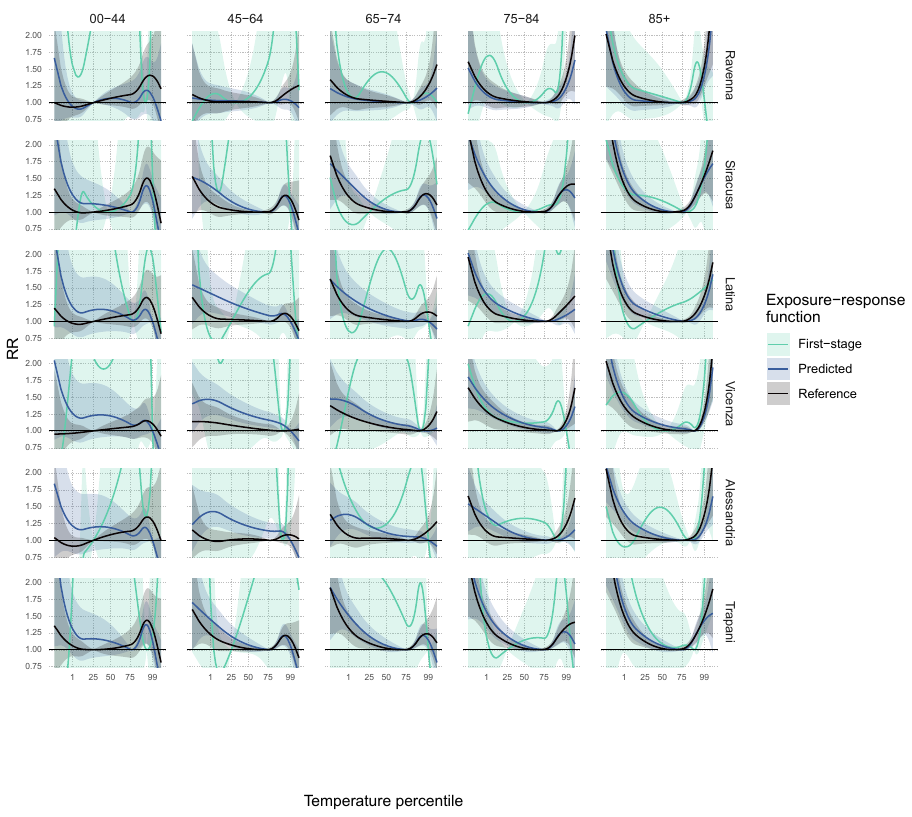


Figure S3: *Age-group and city-specific first-stage, predicted and reference exposure-response functions for the 27 unobserved cities. “First-stage” indicates estimate from a location and age-specific model applied on these cities’ mortality series, “Predicted” is the prediction from the framework, and “Reference” corresponds to the BLUP from a meta-regression model hat includes the unobserved cities.*

# References

1. Sera F, Armstrong B, Blangiardo M, et al. An extended mixed-effects framework for meta-analysis. *Stat Med* 2019; 38: 5429–5444.

2. Burnham KP, Anderson DR. Multimodel Inference: Understanding AIC and BIC in Model Selection. *Sociol Methods Res* 2004; 33: 261–304.

3. Matheron G. Principles of geostatistics. *Econ Geol* 1963; 58: 1246–1266.

4. Hoek G, Beelen R, de Hoogh K, et al. A review of land-use regression models to assess spatial variation of outdoor air pollution. *Atmos Environ* 2008; 42: 7561–7578.

5. Ignaccolo R, Mateu J, Giraldo R. Kriging with external drift for functional data for air quality monitoring. *Stoch Environ Res Risk Assess* 2014; 28: 1171–1186.
